# The Effect of Polymer Content on the Non-Newtonian Behavior of Acetaminophen Suspension

**DOI:** 10.1155/2013/907471

**Published:** 2013-09-10

**Authors:** Eskandar Moghimipour, Maryam Kouchak, Anayatollah Salimi, Saeed Bahrampour, Somayeh Handali

**Affiliations:** ^1^Cellular and Molecular Research Center, Ahvaz Jundishapur University of Medical Sciences, Ahvaz 61357-33184, Iran; ^2^Nanotechnology Research Center, Ahvaz Jundishapur University of Medical Sciences, Ahvaz 61357-33184, Iran; ^3^Department of Pharmaceutics, Faculty of Pharmacy, Ahvaz Jundishapur University of Medical Sciences, Ahvaz 61357-33184, Iran

## Abstract

Acetaminophen is used as an analgesic and antipyretic agent. The aim of the study was evaluation of the effect of different polymers on rheological behavior of acetaminophen suspension. In order to achieve controlled flocculation, sodium chloride was added. Then structural vehicles such as carboxymethyl cellulose (CMC), polyvinyl pyrrolidone (PVP), tragacanth, and magnesium aluminum silicate (Veegum) were evaluated individually and in combination. Physical stability parameters such as sedimentation volume (*F*), redispersibility (*n*), and growth of crystals of the suspensions were determined. Also, the rheological properties of formulations were studied. The results of this study showed that the combination of suspending agents had the most physical stability and pseudoplastic behavior with some degree of thixotropy. Viscosity of suspensions was increased by adding NaCl 0.02%. Presence of PVP is necessary for improving rheological behavior of suspensions by NaCl. This may be related to the cross-linking between the carbonyl group in the PVP segment and Na^+^ ions.

## 1. Introduction

A suspension is a dispersed system in which the internal phase consists of solid particles and the external phase is a liquid vehicle. Suspensions are the best conventional liquid dosage forms of drugs with high bioavailability in comparison to other dosage forms except solutions, and they have patient compliance [1, 2]. Rheological study of suspensions provides valuable information for efficient utilization, transport, and handling of materials in industrial applications [3]. The thixotropy and hysteresis loop are rheological phenomena. In non-Newtonian systems if the rate of shear was reduced once the desired maximum rate had been reached, the down curve can be displaced relative to the up curve. With pseudoplastic systems, the down curve is frequently displaced to the left of the up curve. This phenomenon, known as thixotropy, can be defined as an isothermal and comparatively slow recovery, on standing of a material, which has lost its consistency through shearing [4, 5]. The area surrounded between ascending and descending curves that is called hysteresis loop can give information about the structure breakdown and rebuilding [4, 6, 7]. Controlled flocculation and rheologic modification are important factors in preparation of suspensions. Flocculated suspensions are settled rapidly to form large loose and easily dispersible sediments [8]. Non-Newtonian polymers are utilized in the industries such as food, textile, pharmaceutical, and cosmetics. They are employed in suspensions as structural vehicles and exhibit non-Newtonian (plastic or pseudoplastics) flow with some degree of thixotropy. Various types of polymers are used as rheology control agents such as CMC, methylcellulose, NaCMC, PVP, xanthan gum [6, 9–11], poloxamer [12], tragacanth [13], chitosan [6], and Veegum [14].

Acetaminophen is an analgesic and antipyretic agent whose oral delivery especially to children is combined with trouble due to bitter and unpleasant taste. One of the methods to achieve the maximum taste masking characteristic is to formulate the drug in suspension form which creates a physical fence around the drug [15, 16]. The objective of this work was to formulate acetaminophen as a relatively stable liquid form and study the influence of different polymers such as CMC, Veegum, tragacanth, and PVP on its rheologic characteristics.

## 2. Experimental

Polysorbates 80, sodium chloride, carboxymethyl cellulose (CMC), polyvinyl pyrrolidone (PVP), tragacanth, and magnesium aluminum silicate (Veegum) were purchased from Merck, Germany. Acetaminophen was kindly donated by Chemidarou Pharmaceutical Co., Iran.

### 2.1. Preparation of Suspensions

Finely powdered (120 mesh) acetaminophen (3.2%) was used to prepare suspensions using Veegum (2%), CMC (0.5%), PVP (1%), or tragacanth (0.75%) alone and their different combinations as structural vehicles (Table 1). Polysorbate 80 (0.35%) and sodium chloride (0.02 and 0.04%) were added as wetting and flocculating agents, respectively. Then physical stability and rheological properties of the formulations were evaluated.

### 2.2. Physical Stability

After preparation, sedimentation volume (*F*) of the suspensions was measured daily, and heights of sediments were measured when there was no change in 3 consecutive readings. In order to evaluate the ease of redispersion, suspension samples were rotated periodically at 180 degree. The number of revolutions (*n*) was recorded when the suspension restored to homogeneity [2]. The crystal growth acetaminophen in different suspensions that were stored two months at room temperature was examined by optical microscope (Olympus, R4, Japan).

### 2.3. Rheological Assessment

Rheological behavior of the acetaminophen suspensions was determined using a Brookfield viscometer (Dial reading LVT, USA with no. 3 spindle). Viscosity of samples was determined at 0.3, 0.6, 1.5, 3, 6, 12, 30, and 60 rpm after 1 min rotation at the room temperature. The results were plotted as rheograms and their rheological behaviors were determined by fitting on the corresponding Newtonian and non-Newtonian equations (1):

(1) $$\begin{array}{c} \tau^{N}=\eta^{\prime }\delta ,\\ \text{Log}\;\delta =N\log\tau -\log\eta^{\prime }, \end{array}$$

where *τ* is shear stress, *δ* and *η*′ are shear rate and viscosity coefficient, respectively. *N* is an indicator for defining the type of flow. Since the viscosity of pseudoplastic substances decreases with increasing rate of shear, the apparent viscosity of the formulations at shear rates corresponding to 30 rpm was obtained from the slope of the tangent to the curve at that point. The area of the hysteresis loop of the rheograms can be calculated from the difference between the areas under the up curve and the down curve by using the trapezoidal rule [4, 6, 7].

## 3. Results and Discussion

Comparison of the sedimentation volume in acetaminophen suspension without any suspending agent (formulation *F*_1_) with those suspensions containing one kind of structural vehicle showed that increasing tragacanth and Veegam could increase the sedimentation volume considerably (Table 2). The highest and the lowest sedimentation volumes were observed in suspensions containing tragacanth (86.5 ± 1.25%) and PVP (13 ± 0.82%), respectively. So Veegum (2%) and tragacanth (0.75%) were used in all blend formulations, but CMC and PVP in different formulations were changed. Nag in 2005 studied the stability and flow behavior of barium sulphate suspensions in the presence of various polymers such as PVP. Results showed that PVP had no significant effect on sedimentation volume [10]. *F* and *n* values of the suspensions of different structural vehicles and flocculating agent are shown in Table 2. The suspending agents alone were not able to suspend particles, while their combination showed excellent results. According to the results of the ease of redispersion, formulations *F*_8_ and *F*_11_ with the concentration of 0.04% NaCl were not able to disperse ideally. 

The values of *N* as an indicator for defining the type of flow for different formulations are presented in Table 3. In Newtonian fluids, shear stress and shear rate are directly proportional (*N* = 1), so the rheogram will be a straight line, while, in non-Newtonian fluids, there is not a direct relationship between them (*N* > 1) [17]. Dilatant systems are inverse of that possessed by pseudoplastic systems (*N* < 1) [4]. According to the values of *N*, all formulations showed pseudoplastic behavior. The important parameter for predicting flow behavior of liquid dispersion is the area of the hysteresis loop, which is shown in Table 3. Evaluation of hysteresis area revealed that all of the formulations except formulations *F*_8_ and *F*_11_ had thixotropy behavior. It is generally accepted that greater hysteresis area leads to stronger thixotropic property, and a good suspension should have a relatively high pseudoplastic behavior and some degree of thixotropy [18].

**Figure 1:**
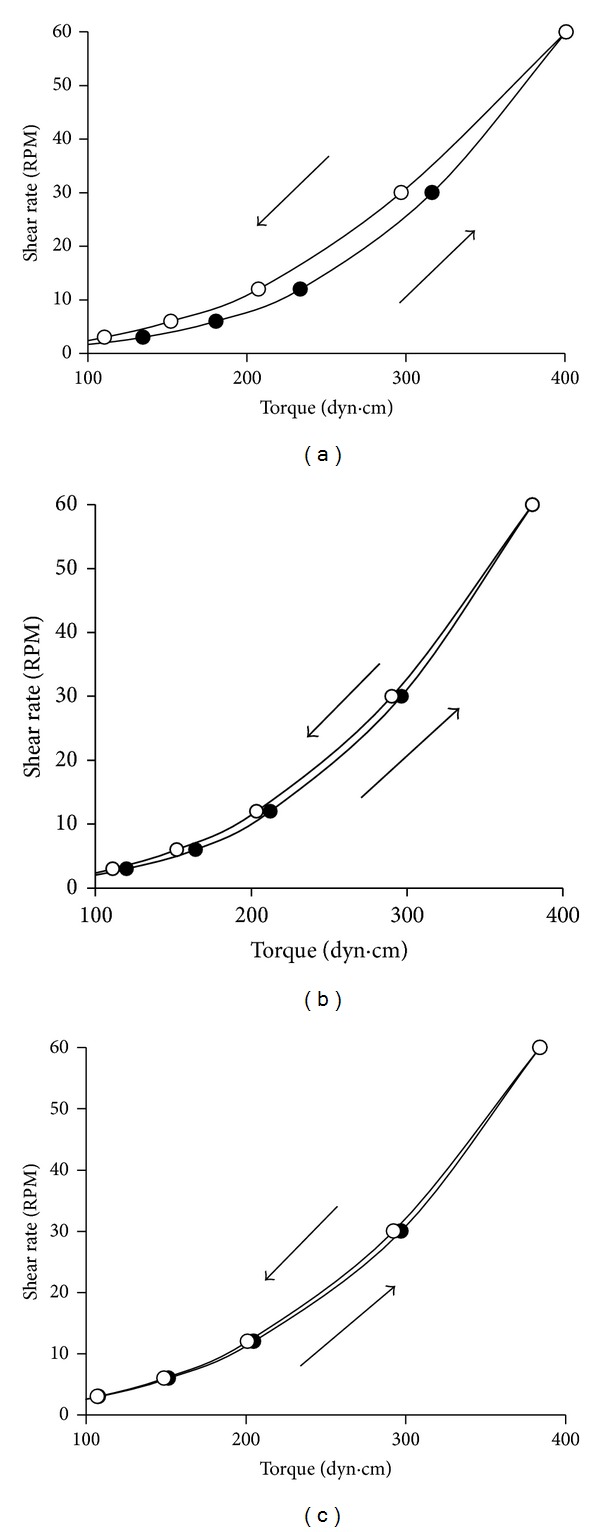
Rheograms and thixotropy of acetaminophen suspensions in formulations (a) *F*_7_, (b) *F*_10_, and (c) *F*_13_.

In formulations *F*_9_–*F*_11_ all suspending agents were used. The value of hysteresis loop and apparent viscosity in formulation *F*_9_ without NaCl were 279.9 dyne · cm · min^−1^ and 564.05 cp, respectively (*P* < 0.05). At low concentrations of NaCl (0.02%), the value of hysteresis loop and apparent viscosity of formulation *F*_10_ increased (Figure 1 and Table 3) (*P* < 0.05). In formulation *F*_11_ with high concentration of NaCl (0.04%), the apparent viscosity of suspension was drastically rinsed so that the instrument could not show any value for torque.

Regarding above mentioned results, presence of NaCl in formulations *F*_10_ and *F*_11_ increased the apparent viscosity in comparison with formulation *F*_9_ (without NaCl). Suspension *F*_6_ is the same as *F*_9_, but it did not contain CMC in its formulation. The value of hysteresis loop and apparent viscosity of *F*_6_ was 286 dyne · cm · min^−1^ and 290.93 cp, respectively. But with adding NaCl (0.02%) in formulation *F*_7_, the value of hysteresis loop and apparent viscosity increased (984 dyne · cm · min^−1^ and 635.30 cp). Formulation *F*_7_ had the highest hysteresis loop in comparison with other formulations (Figure 1(a)). As with formulation *F*_11_, in formulation *F*_8_, with increasing concentration of NaCl (0.04%), the apparent viscosity of the suspension was too high to be detected by the instrument.

Comparison of formulations *F*_7_ and *F*_10_ showed_,_ when NaCl was added as flocculating agent, presence of CMC (formulation *F*_10_) caused a decrease in the value of hysteresis loop.

The value of hysteresis loop and apparent viscosity in formulation *F*_12_ without NaCl and PVP was 486.9 dyne · cm · min^−1^ and 831.23 cp, respectively. But by adding NaCl, in formulation *F*_13_ without PVP, the area of hysteresis loop decreased to 157 dyne · cm · min^−1^ (Figure 1(c)), and the value of apparent viscosity was 670.92 cp. In formulation *F*_13_ without PVP, NaCl not only could not increase the hysteresis loop and viscosity, but also these values were less than those in formulation *F*_12_. The results of rheological assessment indicated that, when NaCl (0.02%) is added as flocculating agent, additional PVP may be necessary for improving thixotropy. Flocculating agents are added to reduce the electrical forces of repulsion between particles and to allow flocks to be formed in order to prevent cake formation [9]. It can be suggested that enhancement of thixotropy and viscosity in formulations containing NaCl and PVP may be related to the cross-linking between the carbonyl group in the PVP segment and Na^+^ ions [19], which partially prohibits the free mobility of the molecular segment and finally results in improvement of the apparent viscosity. Hao et al. in 2007 investigated the rheological behavior of PVP in N,N-dimethylformamide solutions containing metal chlorides (LiCl, CaCl_2_, and CoCl_2_) [19]. The results showed the apparent viscosity of the PVP solutions increased with increasing metal-ion concentration. NMR spectroscopy showed that there were interactions between the metal ions and the carbonyl groups of the PVP segments in the N,N-dimethylformamide solutions, which partially prohibits free mobility of the molecular segment. Also, DSC results indicated that the glass transition temperatures of the PVP/metal chloride composites increased with the addition of metal ions [19].

**Figure 2:**
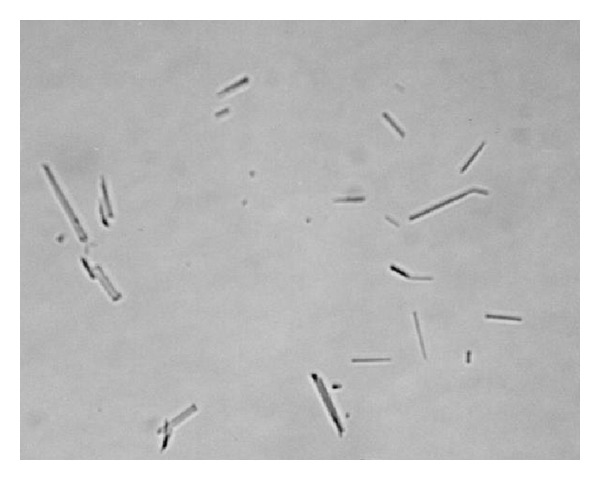
Microscopic view of crystal growth in acetaminophen suspension (*F*_6_) (magnification ×40).

In spite of above results, it is well known that using hydrophilic gums such as PVP and gelatin and polysorbates leads to their adsorption at particle surface and retards crystal growth [9]. Nevertheless, microscopic observations showed the growth of crystals in all formulations of acetaminophen suspensions (as shown in suspension *F*_6_ in Figure 2). It can be hypothesized that changing the amount of factors such as PVP and polysorbate factors in the formulation of the suspensions will prevent crystal growth. 

## 4. Conclusion

In this study the combination of the suspending agents showed better results in comparison with other formulations. Rheological studies showed pseudoplastic behavior for all suspensions prepared by combination of the suspending agents. NaCl 0.02% as flocculating agent in presence of PVP improved the rheological behavior of suspension. 

## Figures and Tables

**Table 1:** Composition of different formulations of acetaminophen suspensions.

Formulation	Tragacanth (%)	Veegum (%)	CMC (%)	PVP (%)	NaCl (%)
*F*_1_	0	0	0	0	0
*F*_2_	0.75	0	0	0	0
*F*_3_	0	0	0.5	0	0
*F*_4_	0	2	0	0	0
*F*_5_	0	0	0	1	0
*F*_6_	0.75	2	0	1	0
*F*_7_	0.75	2	0	1	0.02
*F*_8_	0.75	2	0	1	0.04
*F*_9_	0.75	2	0.5	1	0
*F*_10_	0.75	2	0.5	1	0.02
*F*_11_	0.75	2	0.5	1	0.04
*F*_12_	0.75	2	0.5	0	0
*F*_13_	0.75	2	0.5	0	0.02

**Table 2:** The value of sedimentation volume (*F*) and ease of redispersion (*n*) for acetaminophen suspension in different formulations (mean ± SD *n* = 4).

Formulation	*F* (%)	*n*
*F*_1_	10 ± 0.82	6 ± 0.00
*F*_2_	86.5 ± 1.25	4 ± 0.00
*F*_3_	23.5 ± 2.2	10 ± 1.40
*F*_4_	31 ± 0.96	2 ± 0.00
*F*_5_	13 ± 0.82	3 ± 0.00
*F*_6_	97 ± 1.1	4 ± 0.82
*F*_7_	97 ± 0.48	4 ± 0.50
*F*_8_	97 ± 0.82	∗
*F*_9_	99 ± 1.15	4 ± 0.82
*F*_10_	98 ± 0.25	4 ± 0.00
*F*_11_	98 ± 0.7	∗
*F*_12_	97 ± 1.75	8 ± 1.60
*F*_13_	98 ± 0.82	4 ± 0.0

*Not dispersed after 20 rotations.

**Table 3:** Indicator for defining the type of rheological behavior (*N*), hysteresis loop, and pseudoplastic viscosity at 30 rpm (*η*_30_) in different formulations.

Formulation	*N*	Hysteresis loop (dyne·cm/min)	*η*_30_ (cp)
*F*_6_	2.039	286	290.93
*F*_7_	2.063	984	635.30
*F*_8_	∗	∗	∗
*F*_9_	1.973	279.9	564.05
*F*_10_	2.030	327.9	635.30
*F*_11_	∗	∗	∗
*F*_12_	2.081	486.9	831.23
*F*_13_	1.860	157	670.92

*More than instrument detection range.
